# Pediatric immune myelofibrosis (PedIMF) as a novel and distinct clinical pathological entity

**DOI:** 10.3389/fped.2022.1031687

**Published:** 2022-11-07

**Authors:** Fabiola Guerra, Vincenzo L’Imperio, Sonia Bonanomi, Marco Spinelli, Tiziana Angela Coliva, Fabiola Dell’Acqua, Giulia Maria Ferrari, Paola Corti, Adriana Balduzzi, Andrea Biondi, Fabio Pagni, Francesco Saettini

**Affiliations:** ^1^ Pediatric Hematology Department, Fondazione MBBM, University of Milano Bicocca, Monza, Italy; ^2^ Tettamanti Research Center, University of Milano-Bicocca, University of Milano Bicocca, Monza, Italy; ^3^ Pathology, Department of Medicine and Surgery, ASST Monza, San Gerardo Hospital, University of Milano-Bicocca, Monza, Italy; ^4^ Department of Pediatrics, University of Milano-Bicocca, European Reference Network (ERN) PaedCan, EuroBloodNet, MetabERN, Fondazione MBBM/Ospedale San Gerardo, Monza, Italy

**Keywords:** myelofibrosis, bone marrow, reticulin fibrosis, autoimmune myelofibrosis, inborn errors of immunity, pediatric immune myelofibrosis

## Abstract

Myelofibrosis is a rare myeloproliferative disorder. The detailed descriptions of myelofibrosis in children and adolescents is limited to a few case series and case reports describing fewer than 100 patients, thus suggesting the extreme rarity of this condition prior to adulthood. Though pediatric patients rarely present the typical features and outcomes usually observed in older people, pediatric myelofibrosis is not considered an independent entity. Here we aim to describe patients with pediatric myelofibrosis, showing different clinical and pathological features when compared to the World Health Organization 2016 Primary Myelofibrosis classification. We retrospectively collected and analyzed 14 consecutive pediatric myelofibrosis diagnosed in our Pediatric hematology outpatient clinic over a six-year period. According to clinical data and bone marrow biopsy findings, patients were classified into three subgroups: adult-like myelofibrosis, pediatric immune myelofibrosis, idiopathic myelofibrosis. Pediatric Immune Myelofibrosis was the predominant subgroup in our cohort (7/14). Pediatric Immune Myelofibrosis is characterized by peculiar bone marrow features (i.e., T lymphocyte infiltration) and a milder course compared to the other patients Pediatric Immune Myelofibrosis is a novel and distinct pathological entity. We suggest to carefully consider Pediatric Immune Myelofibrosis in case of bone marrow biopsies showing myelofibrosis that do not fulfill WHO criteria.

## Introduction

Myelofibrosis (MF) is a clonal myeloproliferative condition, characterized by dysregulated proliferation of megakaryocytes, myeloid and erythroid cells, extramedullary hematopoiesis associated with reactive bone marrow (BM) fibrosis, osteosclerosis, angiogenesis, extramedullary hematopoiesis and abnormal cytokine expression (1). MF can occur *de novo* (primary MF or PMF) or secondary to the development of marrow fibrosis after polycythemia vera or essential thrombocythemia (secondary MF) or in association with chronic myeloid leukemia (2, 3). In 2003, Pullarkat et al. described “primary autoimmune myelofibrosis” (AIMF) as MF occurring in patients presenting autoimmune (AI) biological signs in the absence of a well-defined autoimmune disease (AID). Conversely, the term “secondary AIMF” was used for MF occurring with a well-defined AID (4).

MF and particularly PMF in children are exceedingly rare. The detailed descriptions of children and adolescents with PMF and AIMF are limited to a few case series and case reports describing fewer than 100 patients (5–8), thus suggesting the extreme rarity of this condition. According to WHO 2016, the diagnosis of PMF (pre-PMF and overt PMF) requires all three major criteria and at least one minor criterion. The Major criteria include: Megakaryocytic proliferation and atypia, without reticulin fibrosis >grade 1, accompanied by increased age-adjusted BM cellularity, granulocytic proliferation and often decreased erythropoiesis(pre-PMF)/Megakaryocyte proliferation and atypia accompanied by either reticulin and/or collagen fibrosis (grade 2 or 3) (overt PMF); Not meeting WHO criteria for BCR-ABL1 + CML, PV, ET, MDS, or other myeloid neoplasm; Presence of JAK2, CALR, or MPL mutation or in the absence of these mutations, presence of another clonal marker or absence of minor reactive BM reticulin fibrosis. The Minor criteria include one or more of these findings, confirmed in two consecutive determinations: Anemia not attributed to a comorbid condition; Leukocytosis ≥11 × 10^9^/L; palpable splenomegaly; LDH level above the upper limit of the institutional reference range; Leukoerythroblastosis (only for overt PMF) (9). Despite pediatric patients rarely show the typical features and outcomes observed in PMF, the World Health Organization (WHO) 2016 classification has not defined Pediatric Myelofibrosis (PedMF) as an independent entity (9). These lack of data on PedMF implies the absence of a consensus regarding proper treatment and management of PedMF.

We describe the characteristics and outcomes of fourteen consecutive patients with PedMF treated in our center from 2015 to 2021. Clinical, histological, and genetic features of PedMF are quite different compared with those reported in adult MF. Our results show that MF may occur in children with conditions such as autoimmune disorders, inborn error of immunity (IEI, not yet described as associated with MF), and other heterogeneous molecular causes, which we collectively define as Pediatric Immune Myelofibrosis (PedIMF). Such a pattern was the most frequent form of PedMF and laboratory, clinical, histological data and need for treatment reveal that PedIMF may be considered a distinct clinical pathological entity.

## Methods

**Figure 1:**
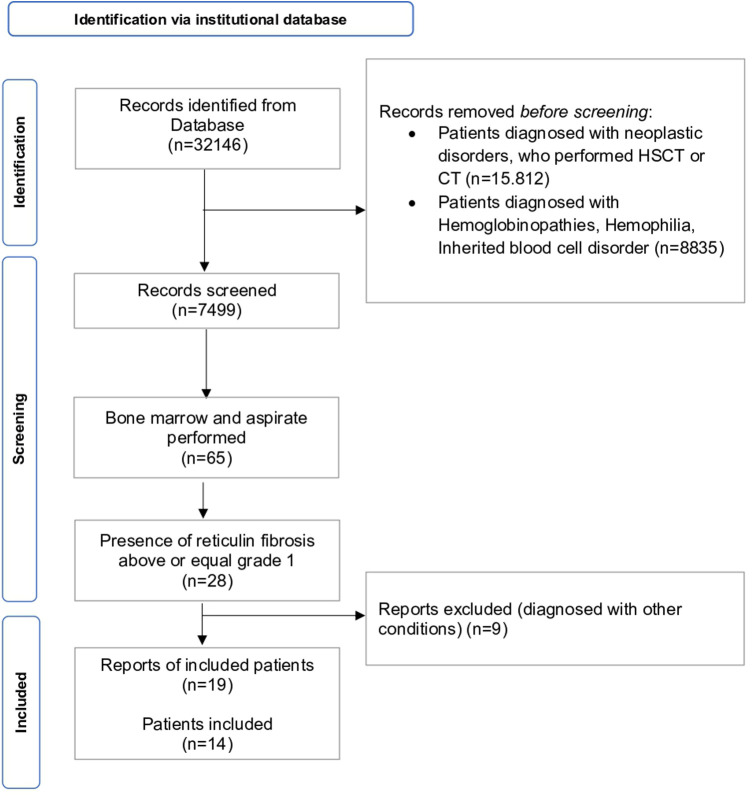
PRISMA flowchart of patients’ retrieval, inclusion and exclusion of records. CT = chemotherapy, HSCT = hematopoietic stem cell transplantation.

We performed a retrospective analysis (Figure 1) of patients' examinations performed in our Pediatric Hematology Unit between January 2015 and December 2021 (*n* = 32,146) screening our institutional database. Patients' examinations were included if one or more of the following inclusion criteria were satisfied: (i) patients referred for isolated or multiple cytopenia (anemia, thrombocytopenia, neutropenia, leukopenia), thrombocytosis, erythrocytosis, leukocytosis, eosinophilia, splenomegaly; (ii) BM biopsy performed between January 1^st^, 2015–December 31^st^, 2021; (ii) presence of reticulin fibrosis above or equal to grade 1 [sec. WHO 2016 (9)]. The exclusion criteria were as follows: (i) patients who had hematopoietic stem cell transplantation (HSCT), or chemotherapy (ii) patients diagnosed with hemoglobinopathies, inherited blood cell disorder (i.e., Fanconi anemia, Blackfan Diamond Anemia, hereditary spherocytosis) or neoplastic disorders.

We collected the reports from screened patients' examinations (*n* = 7,499). We selected patients who performed BM biopsy (*n* = 65) and they were included if BM presented with reticulin fibrosis above or equal to grade 1 (*n* = 28). Nine records were excluded as diagnosed with other conditions (i.e., SAMD9L syndrome, *n* = 1; BMF due to *DNAJC21* variants, *n* = 2; Hoyeraal-Hreidarsson syndrome, *n* = 1; hemochromatosis, *n* = 1; essential thrombocythemia *n* = 2, aplastic anemia, *n* = 2). Finally, a cohort composed of 14 patients performing 18 BM threphine biopsies (four individuals performed an additional BM biopsy each) was identified. Medical records and complete blood counts, peripheral blood (PB) smears, hepatic and renal function tests, BM aspirate and biopsy findings, cytogenetics and molecular profile were collected and analyzed. Infections, AI disorders, endocrine work-up and malignancies were also evaluated. BM biopsies were reviewed for histopathologic features of MF including cellularity, vascularity, osteosclerosis megakaryocytic hyperplasia and clustering, dysmegakaryopoiesis, myeloid hyperplasia, myeloid to erythroid ratio, morphologic dysplasia and immunohistochemical staining. Immunophenotyping (CD34, CD117, CD20, CD3) was performed in case of immune disorder or autoantibodies positivity. Fibrosis was graded as normal, mild, moderate, or severe (MF-0 to MF-3), according to the consensus criteria of Thiele et al. (10). BM biopsies were examined in parallel by two hemopathologists (F.P., V.L.) and minor discordances during the evaluation were solved by consensus. Molecular analyses were performed in eleven out of fourteen patients, following clinical decisions on a case-by-case basis (Table 1). Targeting next generation sequencing panels are detailed in Supplementary Tables S1, S2. For continuous variables, mean and standard deviation (SD) or quartiles (Q1, median and Q3), as appropriate, were calculated, while qualitative variables were reported as count and frequency. Chi-square and ANOVA tests were used to compare dichotomous and continuous variables, respectively. Included patients were classified as follows:

• “Adult-like PMF” in case of patients who either met all the WHO 2016 criteria or presented the typical features of PMF (with or without the presence of molecular lesions) (9);
• “Pediatric Immune MF” (PedIMF) in case of patients presenting with AI predisposing syndromes (11), autoantibodies without defined disorders (4) or known IEI (12);
• idiopathic MF in those not fulfilling the above defined criteria.

**Table 1:** Molecular analysis performed in the included patients (*n* = 14).

Analysis	Number of investigated patients (%)
*JAK2* V617F	11/14 (78%)
*MPL* exon 10	3/14 (21%)
*CALR* exon 9	3/14 (21%)
FHL associated genes	1/14 (7.1%)
*GATA2*	1/14 (7.1%)
NGS panel A	7/14 (50%)
NGS panel B	5/14 (35.7%)
Whole exome sequencing	1/14 (7.1%)

## Results

### Clinical and laboratory profile

Clinical and laboratory data of the 14 included patients are summarized in Tables 2, 3. Seven patients being female and seven male, respectively. The median age at the time of diagnosis was 13 years old (range 1–18 years). There was no family history of hematological disorders except for one patient whose father and sibling were affected by anemia and thrombocytopenia, even though those relatives were not available for further investigations. Two patients presented with a family history of solid malignancies (melanoma, thymoma, testicular cancer) or AI thyroiditis each. Systemic symptoms were rarely reported. At the time of the first clinical evaluation, four patients had splenomegaly. None had a history of severe, recurring or acute infections. No patient had jaundice, lymphadenopathy, gout, bone pain, night sweats, bleeding, congestive cardiac failure, renal dysfunction, thrombosis, portal hypertension or hepatic failure. The most common cytopenia was anemia (*n* = 7, mean hemoglobin 10.9 g/dl; range, 3.7–16.3 g/dl) followed by thrombocytopenia (*n* = 3, mean platelet counts 53 × 10^9^/L, range 7–70 × 10^9^/L). One patient had pancytopenia. Two patients had thrombocytosis (mean platelet count 1019 × 10^9^/L, range 146–1892 × 10^9^/L). The mean leucocyte count was 10.300/mm^3^. One individual had eosinophilia.

**Table 2:** Clinical and presenting features of enrolled patients (*n* = 14). ASA, acetylsalicylic acid; FH Syndrome, familial hemophagocytic lymphohistiocytosis (FHL) syndrome.

	Age (years) or number of patients (%)
**Age**	
Median	13 years, IQR: 8 years
**Gender**	
Male	*n* = 7 (50%)
Organomegaly	*n* = 5 (35.7%)
**Blood count**	
Anemia	*n* = 7 (50%)
Thrombocytopenia	*n* = 3 (21.4%)
Thrombocytosis	*n* = 2 (14.3%)
Pancytopenia	*n* = 1 (7.1%)
Eosinophilia	*n* = 1 (7.1%)
**Genetic abnormalities**	
Trisomy 21	*n* = 2 (14.3%)
JAK2V617 mutation	*n* = 1 (7.1%)
CALR	*n* = 1 (7.1%)
GATA2	*n* = 1 (7.1%)
STXBP2	*n* = 1 (7.1%)
**Reticulin Fibrosis**	
MF-1	*n* = 6 (42.9%)
MF-2	*n* = 6 (42.9%)
MF-3	*n* = 2 (14.3%)
**Bone marrow**	
Hypercellular	*n* = 5 (35.7%)
Normocellular	*n* = 6 (42.9%)
Hypocellular	*n* = 3 (21.4%)
Megakaryocytic hyperplasia	*n* = 7 (50%)
Megakaryocytic clustering	*n* = 5 (35.7%)
Dysmegakaryopoiesis	*n* = 11 (78.6%)
Intrasinusoidal hematopoiesis	*n* = 4 (28.6%)
Lymphocytic infiltration	*n* = 5 (35.7%)
Osteosclerosis	*n* = 0
**Pediatric Immune Myelofibrosis**	
Sjogren syndrome	*n* = 1 (7.1%)
GATA2 deficiency	*n* = 1 (7.1%)
FHL syndrome	*n* = 1 (7.1%)
Down syndrome	*n* = 2 (14.3%)
Autoimmune neutropenia	*n* = 1 (7.1%)
L-HES	*n* = 1 (7.1%)
**Treatment**	
Steroid course	*n* = 2 (14.3%)
ASA treatment	*n* = 2 (14.3%)
RBC and PLT transfusions	*n* = 6 (42.9%)
Chemotherapy	*n* = 1 (7.1%)
HSCT	*n* = 2 (14.3%)
Splenectomy	*n* = 1 (7.1%)
**Outcome and follow-up**	
Median time of follow-up	5.4 years, IQR: 4.25 years
Alive	*n* = 14 (100%)

IQR, interquartile range; FHL, familial haemophagocytic lymphohistiocytosis; HSCT, hematopoietic stem cell transplantation; MF-1, reticulin fibrosis grade 1; MF-2, reticulin fibrosis grade 2; MF-3, reticulin fibrosis grade 3; RBC, red blood cells; PLT, platelets.

**Table 3:** Comparison between WHO 2016 criteria and pediatric MF criteria of the 14 included patients.

Patient	1	2	3	4	5	6	7	8	9	10	11	12	13	14
**Major criteria WHO 2016**														
Megakaryocytic proliferation and atypia, without reticulin fibrosis >grade 1, accompanied by increased age-adjusted BM cellularity, granulocytic proliferation and often decreased erythropoiesis (prePMF)/Megakaryocyte proliferation and atypia accompanied by either reticulin and/or collagen fibrosis (grade 2 or 3) (overt PMF)	Yes	Yes	Yes	Yes	Yes	Yes	Yes	Yes	Yes	Yes	Yes	Yes	Yes	Yes
Not meeting WHO criteria for BCR-ABL1 + CML, PV, ET, MDS, or other myeloid neoplasm	Yes	Yes	Yes	Yes	Yes	Yes	Yes	Yes	Yes	Yes	Yes	Yes	Yes	Yes
Presence of JAK2, CALR, or MPL mutation or in the absence of these mutations, presence of another clonal marker or absence of minor reactive BM reticulin fibrosis	No	Yes	No	Yes	No	No	No	No	No	No	No	No	No	No
Minor criteria WHO 2016														
Palpable spleen	No	Yes	No	Yes	Yes	No	No	Yes	Yes	No	No	No	No	No
Anemia	No	No	Yes	Yes	Yes	No	Yes	Yes	Yes	Yes	No	Yes	No	No
LDH Increased	Yes	Yes	Yes	Yes	No	No	No	Yes	No	Yes	Yes	–	–	Yes
Leukocytosis >11 × 10^9^/L	No	No	No	No	No	No	No	No	No	No	No	No	No	No
Leukoerythroblastosis	No	No	Yes	Yes	–	–	–	–	–	No	No	–	–	–
**Pediatric Myelofibrosis**														
Proposed PedMF Classification	Adult-like	Adult-like	Adult-like	Adult-like	Idiopathic MF	Idiopathic MF	Idiopathic MF	Immune MF	Immune MF	Immune MF	Immune MF	Immune MF	Immune MF	Immune MF
PedMF criteria	Fulfilling WHO criteria except for molecular markers, BM findings consistent with adult PMF	Fulfilling WHO criteria	Fulfilling WHO criteria	Fulfilling WHO criteria except for molecular markers, BM findings consistent with adult PMF	None	None	None	Autoantibodies positivities (ANA, Coombs); FHL	GATA2 deficiency	Autoantibodies positivities (Lac+), Down's Syndrome	ITP, Down's Syndrome	AIN	Sjiogren's Syndrome	L-HES

AIN, autoimmune neutropenia; BM, bone marrow; CML, chronic myeloid leukemia; ET, essential thrombocythemia; FHL, familial haemophagocytic lymphohistiocytosis; ITP, immune thrombocytopenia; L-HES, lymphocyte-variant hypereosinophilic syndrome; MDS, myelodysplastic syndromes; PMF, primary myelofibrosis; PV, polycitemia vera; WHO, world health organization.

### Classification of pediatric mf

Four patients were classified as having “adult-like PMF” (Pt1–Pt4). Two patients met all the WHO criteria (Pt2 and Pt3), including molecular lesions (*JAK2*, *n* = 1; *CALR*, *n* = 1). A further *ASXL1* variant was detected in one patient (Pt3) who already carried one *CALR* somatic variant. Two patients presented the typical features of PMF without the presence of molecular lesions (Table 3). Increased LDH and leukoerythroblastosis were consistently documented (Pt1–Pt4). Splenomegaly was detected in two patients (Pt2, Pt4). Three out of four patients in this group required treatment. Two children (Pt1, Pt3) needed haematopoietic stem cell transplantation (HSCT) after fully myeloablative conditioning from mismatched unrelated donors. One patient (Pt2), with thrombocytosis and *JAK2* variant, is currently treated with low dose acetylsalicylic acid (ASA).

Three patients (Pt5-Pt7) were classified as idiopathic MF. None of them displayed molecular markers or increased LDH. Two patients (Pt5, Pt7) presented with pancytopenia. Splenectomy was performed in one patient (Pt5) due to massive splenomegaly causing pancytopenia and requiring red blood cell transfusions.

Seven individuals (Pt8–14) were classified as having PedIMF (Table 3). Two patients had IEI, of whom one (Pt9) was diagnosed with GATA2 deficiency when she was 20 years old, while the second one had a concurrent diagnosis of PedIMF and FHL (13). Down's syndrome was diagnosed in two patients (Pt10 and Pt11) in the first year of life. They subsequently developed PedIMF when they were 3 and 14 years old, respectively. One reumatological condition (Siogren Syndrome) was diagnosed in Pt13 at 16 years old. 3 years later, PedIMF was demonstrated. Two patients (pt 12 and Pt 14) presented with hematological conditions (Autoimmune neutropenia and L-HES). BM biopsies showed PedIMF when they were 13 and 10 years old, respectively. Four patients (Pt8–Pt12) had autoantibodies positivity prior to PedIMF. Ana, LAC and Coombs positivity were detected at 7 (Pt8), 2 (Pt10), 5(Pt11) years of age, respectively. PB counts of patients with PedIMF presented with variable features, such as anemia (*n* = 4), thrombocytopenia (*n* = 2), neutropenia (*n* = 1), eosinophilia (*n* = 1) and pancytopenia (*n* = 1). None of them exhibited leukoerythroblastosis. Four children (Pt8, Pt10, Pt11, Pt14) displayed increased LDH. Splenomegaly was detected in two patients (Pt8, Pt10).

### Pathology data

**Figure 2:**
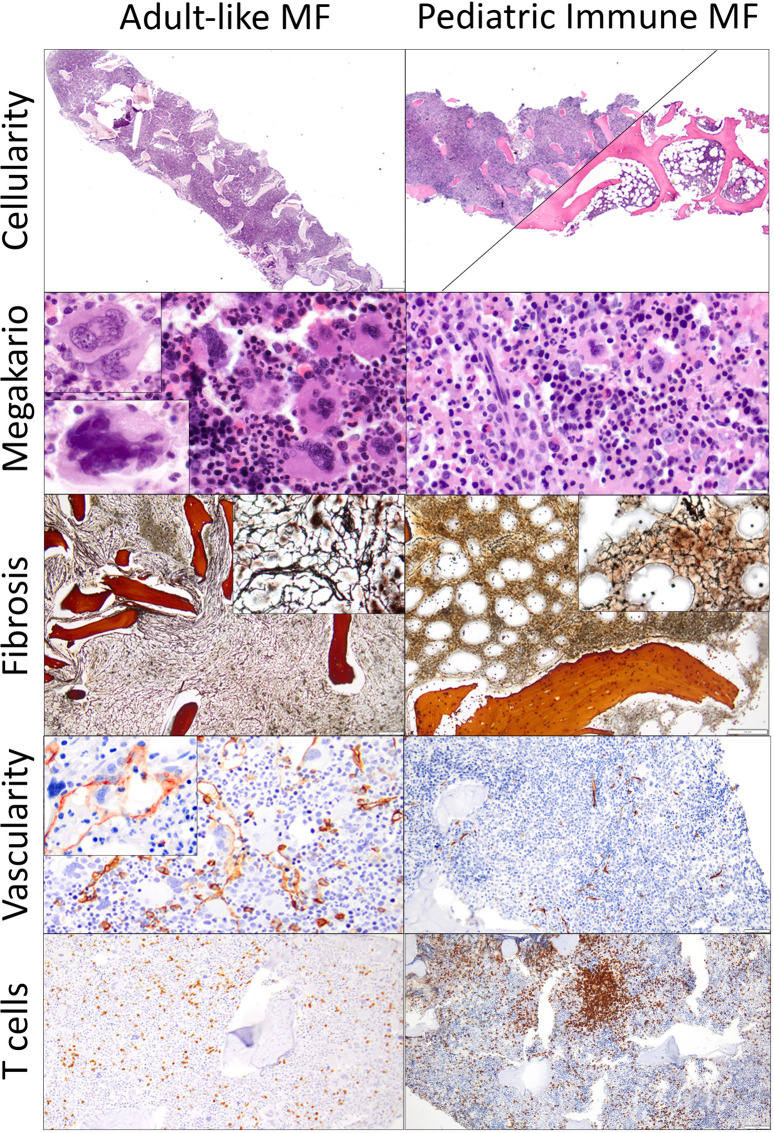
Comparison of the histological features of adult-like MF and PedIMF in this cohort adult-like MF demonstrated higher cellularity (90% in average, **A**, H&E, ×20), frequent clustering of megakaryocytes (**B**, H&E, ×400), with either hyperplastic/bulbous (upper left inset) or dysmorphic (bottom left inset) forms. Moreover, the increase in reticulin was generally high (2 ± 0.82, **C**, H&E, ×100) with frequent thickening and intersection of fibers (upper right inset), with prominent microvascular proliferation (CD34 IHC, **D**, ×200) and occasional intrasinusoidal hematopoiesis (upper left inset) but relatively preserved percentage of infiltrating T lymphocytes (8.3% ± 7.9, CD3 IHC, **E**, ×100). On the other hand, PedIMFshowed a variable cellularity, ranging from 90% to 20% (**F**, H&E, ×20), with almost normal or rarely hyperplastic megakaryocytes (**G**, H&E, ×500), relatively lower increase in reticulin (1.29 ± 0.76, **H**, H&E, ×100) with only occasional intersection of fibers (upper right inset). Microvasculature was often preserved (CD34 IHC, **I**, ×200) and a significant increase in the T lymphocytes infiltration, often arranged in aggregates, was noted in the majority of cases (13.6% ± 7.5, CD3 IHC, **J**, ×100).

BM findings are outlined in Tables 3, 4. The main and most frequent histological differences between Adult-like and PedIMF are reported in Figure 2. Adult-like MF showed an increased cellularity per age (*n* = 3, 75%), with an average of 90% as compared to the adipose component, moderate increase in marrow fibrosis (MF 2 ± 0.82) and prominent vascularity frequently associated with intrasinusoidal hematopoiesis (*n* = 3, 75%) (Table 4, Cellularity and stromal changes section). These cases showed significant hyperplastic changes of megakaryocytes (*n* = 3, 75%), with frequent formation of dense clusters alternating bulbous and dysmorphic elements (Table 4, Megakaryocytes section). On the other hand, PedIMF demonstrated variable cellularity per age, ranging from 20 to 90%, with lower marrow fibrosis on average (MF 1.29 ± 0.76, *p* = 0.029) and an almost preserved vascularity (Table 4, Cellularity and stromal changes section). In idiopathic MF and PedIMF megakaryocytes were normal or only slightly increased, rarely in clusters and with less dysmorphic features (Table 4, Cellularity and stromal changes section). Myeloid hyperplasia was more prominent in the adult-like MF (*n* = 3, 75%) whereas erythroid hyperplasia was more frequent in the idiopathic forms (*n* = 3, 100%) (Table 4, Myeloid and Erythroid hyperplasia section). Finally, no significant increase of the B-lymphocytes was observed, whereas PedIMF cases showed a relatively higher infiltration by T lymphocytes, with PedIMF and idiopathic MF forms showing relatively higher (13.6% ± 7.5 and 11.7% ± 7.6, respectively) percentage of CD3 + cells in the BM biopsy as compared to adult-like MF (8.3% ± 7.9, *p* = 0.029), often with the formation of interstitial aggregates (Table 4, T lymphocytes section).

**Table 4:** Summary of immunePedMF, idiopathic pedMF, adult-like PMF.

	Immune PedMF (*n* = 7)			Idiopathic PedMF (*n* = 3)			Adult-like PMF (*n* = 4)			*p*-value
**Age (y)**										
MEDIAN (years/IQR)	10 years 7 years			4 years 11 years			9 years 8.5 years			
**Gender**										
Male	*n* = 3		42.8%	*n* = 3		100%	*n* = 2		50%	
Female	*n* = 4		57.2%	–			*n* = 2		50%	
**Organomegaly**	*n* = 2		28.6%	*n* = 1		25%	*n* = 2		50%	
**Blood count**										
Anemia	*n* = 3		42.8%	*n* = 2		50%	*n* = 2		50%	
Thrombocytopenia	*n* = 2		28.6%	*n* = 1		25%	–			
Thrombocytosis	–			–			*n* = 2		50%	
Cytopenia	*n* = 1		14.3%	*n* = 1		25%	–			
Eosinophilia	*n* = 1		14.3%	–			–			
**Molecular marker or genetic lesion**										
Normal (*n*, %)	*n* = 3		42.8%	*n* = 3		100%	*n* = 2		50%	
Trisomy 21	*n* = 2		28.6%	–			–			
JAK2V617 mutation	–			–			*n* = 1		25%	
CALR	–			–			*n* = 1		25%	
GATA2	*n* = 1		14.3%	–			–			
STXBP2	*n* = 1		14.3%	–			–			
**Cellularity and stromal changes**										
Cellularity, mean percentage and range	69	%	20%–90%	70	%	50%–80%	90	%	80%–90%	
Hypercellularity per age (*n*, %)	2	,	29%	1	,	33%	3	,	75%	0.304
Myelofibrosis [MF] (mean ± SD)	1.29	±	0.76	2	±	1	2	±	0.82	**0.029**
Increased vascularity and/or intrasinusoidal hematopoiesis (*n*, %)	0			1	,	33%	3	,	75%	**<0.01**
**Megakaryocytes**										
Hyperplasia (*n*, %)	2	,	29%	1	,	33%	3	,	75%	0.304
Clustering (*n*, %)	1	,	14%	0			3	,	75%	**0.021**
Dysmegakaryopoiesis (*n*, %)	4	,	57%	3	,	100%	4	,	100%	0.314
**Myeloid hyperplasia (*n*, %)**	1	,	14%	0			3	,	75%	**0.021**
**Erythroid hyperplasia (*n*, %)**	3	,	43%	3	,	100%	1	,	25%	0.267
**T lymphocytes, CD3+ (mean percentage ± SD and range)**	13.6% ± 7.5		10%–30%	11.7% ± 7.6		5%–20%	8.3%| ± 7.9		5%–20%	**0.029**

### Treatment and follow up

Treatment and follow up data are summarized in Table 2.Median time of follow up at the last visit was 5.4 years (Q25 = 0 years; Q75 = 4.25 years). All patients are alive and well at the time of the last follow up. Ten out of 14 patients are currently followed up in our Pediatric Hematology Outpatients Clinic. Among individuals diagnosed with PedIMF, two required supportive care. Two patients required steroid treatment. Pt14 required a short steroid course, while Pt8 required long-term steroid treatment due to autoimmune hemolytic anemia. Chemotherapy was administered in Pt11 for subsequent diagnosis of acute myeloid leukemia. Three patients (Pt4, Pt11, Pt14) repeated BM aspirate and biopsy and results are shown in Table 5. Cellularity decreased in two out of three patients and reticulin fibrosis in all of them.

**Table 5:** Summary of repeated bone marrow aspirates and biopsies.

Patient	4	8	11	11	14
Molecular marker or genetic lesion	–	STXBP2	Tr.21	Tr.21	–
Cellularity %	50%	90%	80%	90%	50%
Reticulin Fibrosis (MF)	1	2	1	0	1
Increased vascularity	Present	Absent	Absent	Absent	Absent
Intrasinusoidal hematopoiesis	Present	Absent	Absent	Absent	Absent
Osteosclerosis	Absent	Absent	Absent	Absent	Absent
Megakaryocytic hyperplasia	Absent	Absent	Present	Absent	Absent
Megakaryocytic clustering	Absent	Absent	Present	Absent	Absent
Dysmegakaryopoiesis	Present	Present	Absent	Absent	Absent
Palpable spleen	Absent	Absent	Absent	Absent	Absent
Anemia	Absent	Absent	Present	Absent	Absent
Leukoerythroblastosis	–	–	Absent	–	–
LDH Increased	–	Absent	–	Absent	–
Leukocytosis >11 × 10^9^/L	Absent	Absent	Absent	Absent	Absent

## Discussion

Myelofibrosis is characterized by PB cytopenia, leukoerythroblastosis, ineffective hematopoiesis, proliferation of dysfunctional megakaryocytes with reticulin and/or collagen fibrosis in the BM, and dysregulated cytokine production as well as extramedullary hematopoiesis and hepatosplenomegaly (14). A detailed description of Pediatric MF is limited to a few case series focused on PMF (5–7) and AIMF (4, 8). Authors agree that clinical, histological, and molecular features of Pediatric MF differ from adult PMF, but given the rarity of the disease, precise data are lacking. Pediatric MF has a heterogeneous phenotype with variable outcomes ranging from spontaneous resolution to a rapidly progressive, sometimes fatal, disease curable only by HSCT. Indeed, different authors have proposed to consider Pediatic MF as a distinct entity when compared to PMF (14).

We believe that the heterogeneous phenotype of Pediatric MF calls for a classification that includes further subtypes among these patients. Our results seem to identify three defined subgroups of children with MF. The first group includes patients with features that are typically detected in adult patients with PMF (Adult-like PedMF). Laboratory values, commonly seen in adult PMF patients—including increased LDH, bilirubin, and leukoerythroblastosis—all together suggest high marrow cell turnover and are well documented in these children (15). Driver mutations in genes such as *JAK2*, *CALR*, or *MPL* mutations can be detected in the “Adult-like PMF” subgroup. BM hypercellularity, dysmegakaryopoiesis, megakaryocytic hyperplasia, megakaryocytic clustering and myeloid hyperplasia and increased vascularity are common findings. When compared with adult PMF patients reported in the literature, “Adult-like PMF” seems to differ with respect to clinical symptoms. In fact, constitutional symptoms (like fatigue, weight loss, night sweats) are often lacking, despite splenomegaly is present (14). Finally, the need for and the type of treatment administered (i.e., HSCT and ASA) are shared between children with adult-like PMF and PMF.

In the second and most represented group of patients, MF occurs in the context of IEI, known rheumatological or hematological conditions or autoantibodies positivity. We define these children as having PedIMF. In our case series along with predominantly conditions that present with autoantibody and B cell alterations, we expand the concept of immune PedIMF to predominantly T-cell (L-HES) and innate immune defects (GATA2, Familial haemophagocytic lymphohistiocytosis Syndrome). Apart from splenomegaly and increased LDH, which are common findings in PedIMF and are shared with the adult-like cohort, patients with PedIMF present some peculiar features, namely PB results, BM findings and lack of need of treatment. Although PB counts in PedIMF are typically variable, they essentially lack leukoerythroblastosis. These features differentiate PedIMF from Adult-like MF and Idiopathic MF; the latter was mainly characterized by cytopenia. Differences between adult-like MF and PedIMF are evident on the BM examination as well. Indeed, this novel entity is characterized by a variable marrow cellularity, with hypo and hypercellular cases, a relatively lower grade of increase in reticulin, a substantially preserved interstitial vascularity and less prominent myeloid hyperplasia. Another histological hallmark of PedIMF is represented by the prevalence of T lymphocyte infiltration, often organized in central aggregates, stressing the possible link with the underlying autoimmune/IEI disorder (16). In the PedIMF group, in fact a milder course has been observed compared to other groups of our cohort, as already previously reported. Although our series suggests that the majority of MF cases have a benign course with spontaneous resolution, one patient (Pt10) affected by Down Syndrome after being diagnosed with PedIMF, subsequently developed malignant evolution (Acute myeloblastic leukemia). Although the relationship between trisomy 21 and MF has been already described (17, 18) this association can be only speculated due to lack of data.

Idiopathic MF exhibits extreme variability compared to the other groups. Distinctive features are represented by normal level of LDH, absence of megakaryocytic clustering or increased vascularity in BM specimens. Reticulin fibrosis was markedly different, ranging from grade 1 to grade 3. The disease course in the idiopathic PedMF is unpredictable, yet we have to consider the small number of patients.

Given the striking difference in disease course and prognosis between PedIMF and the other entities it is imperative to have reliable diagnostic criteria. Thus, after having re-examined the clinical, laboratory, genetic and morphologic findings of our cohort we highlighted the role of BM T lymphocyte infiltration. Clinicians should be aware that previously unreported AI, immunological, rheumatological conditions may be associated with PedIMF and therefore consider it in the follow-up of these patients. On the other hand, in case of BM infiltrate by T-lymphocytes, AI and IEI workup should be performed in those patients who have no defined diagnosis.

There are some limitations to this study. The number of patients is small, yet the rarity of this disorder has to be taken into account. Genetic testing could have been not uniformly undertaken in all patients due to the retrospective pattern of this analysis. However, our pediatric series characterized three different subgroups of patients, underlining as predominant the immune histopathological and genetic features in a detailed manner, documenting that MF in children is a different entity when compared with adults.

## Conclusions

MF in children have histopathological and genetic features different from adults and it mainly occurs in the context of IEI, known rheumatological or hematological conditions or autoantibodies positivity, collectively we refer to this novel entity as PedIMF. These findings underscore the importance of appropriate diagnostic work-up and treatment of the underlying disorder causing MF.

## Data Availability

The raw data supporting the conclusions of this article will be made available by the authors, without undue reservation.
