# Supplementary material for: Pediatric immune myelofibrosis (PedIMF) as a novel and distinct clinical pathological entity
Source: Front Pediatr. 2022 Nov 7;10:1031687. doi: 10.3389/fped.2022.1031687 (PMC9676962; doi:10.3389/fped.2022.1031687)
Supplement: Supplementary file 1 [file Datasheet1.pdf]

Pediatric Immune Myelofibrosis (PedIMF) as a novel and distinct clinical pathological entity

Fabiola Guerra[1,2], Vincenzo L'Imperio[3], Sonia Bonanomi[1], Marco Spinelli[1], Tiziana Angela Coliva[1], Fabiola Dell'Acqua[1], Giulia Maria Ferrari[1], Paola Corti[1], Adriana Balduzzi[1], Andrea Biondi[4], Fabio Pagni[3], Francesco Saettini[2]

1 Pediatric Hematology Department, Fondazione MBBM, University of Milano Bicocca, Monza, Italy.

2 Centro Ricerca Tettamanti, University of Milano Bicocca, Monza, Italy

3 Pathology, Department of Medicine and Surgery, ASST Monza, San Gerardo Hospital, University of Milano-Bicocca, Monza, Italy.

4 Pediatric Department and Centro Tettamanti-European Reference Network PaedCan, EuroBloodNet, MetabERN-University of Milano-Bicocca-Fondazione MBBM-Ospedale, San Gerardo, Monza, Italy.

*** Correspondence:**
Fabiola Guerra, Pediatric Hematology Department, Fondazione MBBM, University of Milano Bicocca, Via Cadore, 20900, Monza, Italy. Phone +390392333529. Fax: +390392332470. Mail: fabguerra0@gmail.com

Electronic Supplemental Material (ESM)

LIST OF CONTENTS

SUPPLEMENTARY TABLE S1: LIST OF GENES INVESTIGATED WITH NEXT GENERATION SEQUENCING PANELS (PANEL A)

SUPPLEMENTARY TABLE S2: LIST OF GENES INVESTIGATED WITH NEXT GENERATION SEQUENCING PANELS (PANEL B)

Supplementary Table S1

LIST OF GENES INVESTIGATED WITH NEXT GENERATION SEQUENCING PANELS (PANEL A)

ABL1 (4-9)	ASXL1 (9,11,12)	BRAF (15)	CALR (9)	CBL (8,9)	CEBPA (all)
CSF3R (all)	DNMT3A (all)	ETV6 (all)	EZH2 (all)	FLT3 (13-15,20)	HRAS (2,3)
IDH1 (4)	IDH2 (4)	JAK2 (all)	KIT (2,8-11,13,17,18)	KRAS (2,3)	MPL (10)
NPM1 (10,11)	NRAS (2,3)	PTPN11 (3,7-13)	RUNX1(all)	SETBP1 (4)	TET2 (all)

Supplementary Table S2

LIST OF GENES INVESTIGATED WITH NEXT GENERATION SEQUENCING PANELS (PANEL B)

ABCG5	ABCG8	ACTG1	ADAMTS13	AK2	ANKRD26
AP3B1	ASXL1	CD40LG	CECR1	CLPB	CSF3R
CTSC	CXCR2	CXCR4	CYCS	DIAPH1	DNAJC21
EFTUD1	EIF2AK3	ELANE	ETV6	FAS	FASLG
FLI1	FLNA	FNIP1	FYB	G6PC3	GALE
GATA1	GATA2	GATA 2 int.	GFI1	GFI1B	GINS1
GNE	GP1BA	GP1BB	GP9	HAX1	HOXA11
HYOU1	IFNGR2	ITGA2B	ITGB3	JAGN1	KRAS
LAMTOR2	LRBA	LYST	MASTL	MECOM	MKL1
MPL	MYH9	NBEAL2	NRAS	ORAI1	PGM3
PRKACG	PTPRJ	RAB27A	RAC2	RBM8A	RMRPR
RUNX1	SAMD9	SAMD9L	SBDS	SLC37A4	SLFN14
SMARCD2	SRC	SRP54	STIM1	STK4	TAZ
TCIRG1	TCN2	THPO	TPM4	TRPM7	TUBB1
USB1	VPS13B	VPS45	VWF	WAS	WDR1
WIPF1					
